# A review on drones controlled in real-time

**DOI:** 10.1007/s40435-020-00737-5

**Published:** 2021-01-05

**Authors:** Vemema Kangunde, Rodrigo S. Jamisola, Emmanuel K. Theophilus

**Affiliations:** BIUST, Palapye, Botswana

**Keywords:** Drones, Unmanned areal vehicles, Real-time control, Real-time operating system, Global positioning system, Inertial measurement unit

## Abstract

This paper presents related literature review on drones or unmanned aerial vehicles that are controlled in real-time. Systems in real-time control create more deterministic response such that tasks are guaranteed to be completed within a specified time. This system characteristic is very much desirable for drones that are now required to perform more sophisticated tasks. The reviewed materials presented were chosen to highlight drones that are controlled in real time, and to include technologies used in different applications of drones. Progress has been made in the development of highly maneuverable drones for applications such as monitoring, aerial mapping, military combat, agriculture, etc. The control of such highly maneuverable vehicles presents challenges such as real-time response, workload management, and complex control. This paper endeavours to discuss real-time aspects of drones control as well as possible implementation of real-time flight control system to enhance drones performance.

## Introduction

**Fig. 1:**
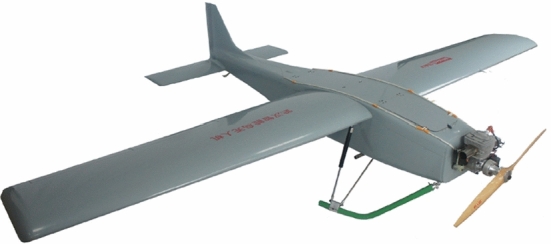
The KC2800 is a fixed-wing drone used for surveillance and mapping Picture reprinted from https://aibirduav.diytrade.com

**Fig. 2:**
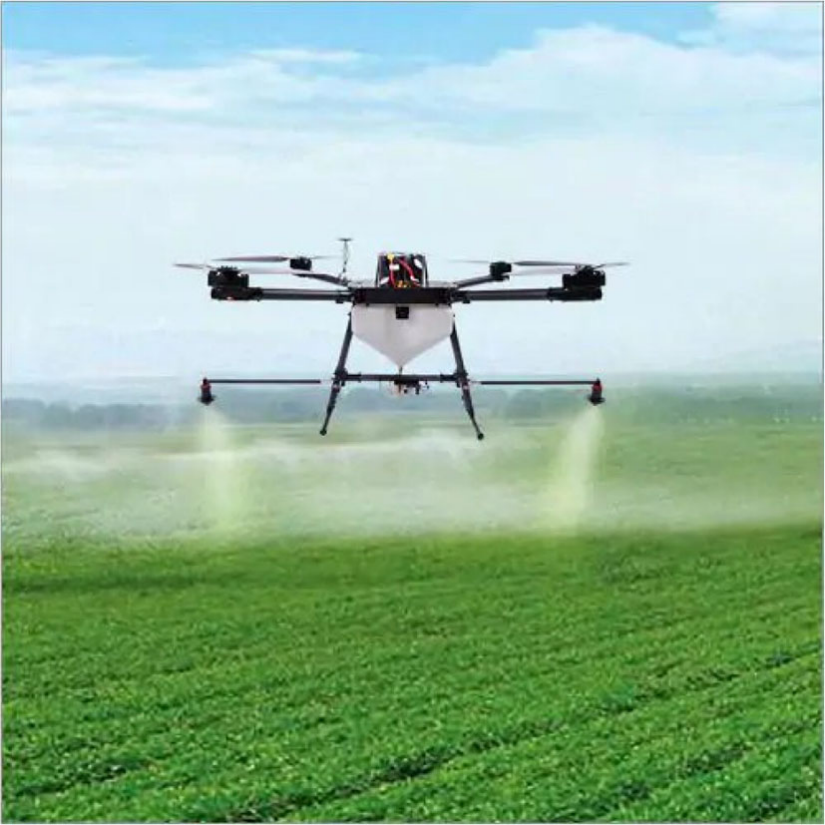
Quadrotor drone spraying pesticide on crops Picture reprinted from https://www.indiamart.com

A drone, also known as unmanned aerial vehicle (UAV), is an aircraft without a human pilot on board [1, 2]. There has been a rapid development of drones for the past few decades due to the advancement of components such as micro electro-mechanical systems (MEMS) sensors, microprocessors, high energy lithium polymer (LiPo) batteries, as well as more efficient and compact actuators [3–5]. Drones are now present in many daily life activities [2, 6–8]. They are used in many applications such as inspecting pipelines and power lines, surveillance and mapping, military combat, agriculture, delivery of medicines in remote areas, aerial mapping, and many others [2, 9–12]. See Figs. 1 and 2 for some drones applications. Robotic manipulators, found in many applications [13–15], have in recent years been implemented on UAV platforms [16–18] for tasks such as aerial manipulation, grasping, and cooperative transportation. The unstable dynamics of the robotic arm, which increase control complexity of UAVs, have widely been studied in the literature [19–22].

UAVs technology is rapidly growing while UAV solutions are being proposed at faster rates as various needs arise. Drone features are determined by specific UAV applications as well as competition in the commercial market [23, 23–25]. In [26], a review of the most recent applications of UAVs in the cryosphere was conducted. Compared to conventional spaceborne or airborne remote sensing platforms [27–29], UAVs offer more advantages in terms of data acquisition windows, revisits, sensor types, viewing angles, flying altitudes, and overlap dimensions [26, 30–32]. The review shows that across the world, applications used various multirotor and fixed-wing UAV platforms. Red, green, blue (RGB) sensors were the most used, and applications utilised quality video transmission to the ground control station. The study in [33] demonstrates how versatile and fast-growing is the adoption of UAV solutions in daily life scenarios. They propose the design of a system capable of detecting coronavirus automatically from the thermal image quickly and with less human interactions using IoT-based drone technology. The UAV system is equipped with two cameras: an optical camera and a thermal camera. It conveys to the ground control station (GCS) the image of the person, the global positioning system (GPS) location as well as a thermal image of the hot body detected. The system combines IoT, virtual reality, and live video feedback to control the camera for monitoring people.

**Fig. 3:**
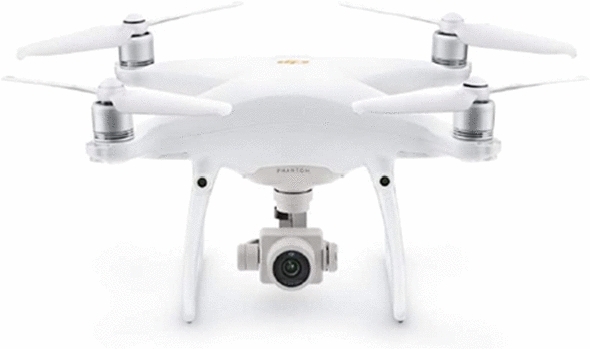
Phantom 4 Picture reprinted from https://thewiredshopper.com

**Fig. 4:**
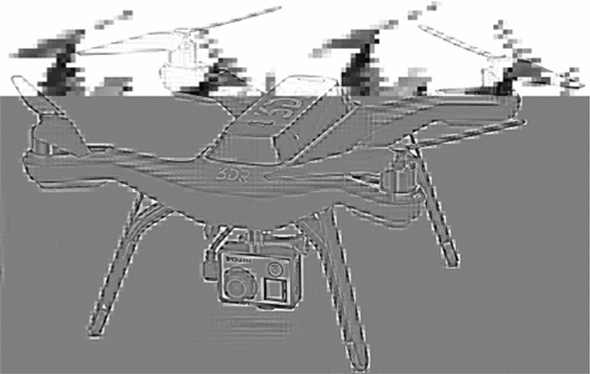
3DR Solo Picture reprinted from https://thewiredshopper.com

On the other hand, apart from advancements in custom-made drones, commercial drone manufacturers are actively improving their products. Latest, more advanced drones are presented at https://thewiredshopper.com, see Figs. 3 and 4. DJI Phantom 4, for example, is equipped with an automatic collision avoidance system. It has a sport mode that disables collision detection and enables fast speeds. It also has an active tracking technology that enables the selection of another moving object, like a car or another drone, and the Phantom 4 will autonomously follow it without assistance from the human pilot. The drone is equipped with a 3-axis camera and can record 4K resolution video at 30 fps and 1080p resolution at 12 fps. It will take 12-megapixel images in Adobe DNG raw format. It has gimbal stabilization technology and a built-in video editor. Other latest drones in the market include the AirDog drone by AirDog, 3DR Solo Drone by 3DRobotics, and Yuneec Typhoon H by Yuneec. A UAV’s operational environment is highly dynamic due to unpredictable changes in weather conditions affecting the air space. For drones to be reliable, their flight controllers must adapt to these environmental changes in real-time. Control of highly maneuverable UAVs has been extensively studied for the past decades.

## Drone hardware overview

**Fig. 5:**
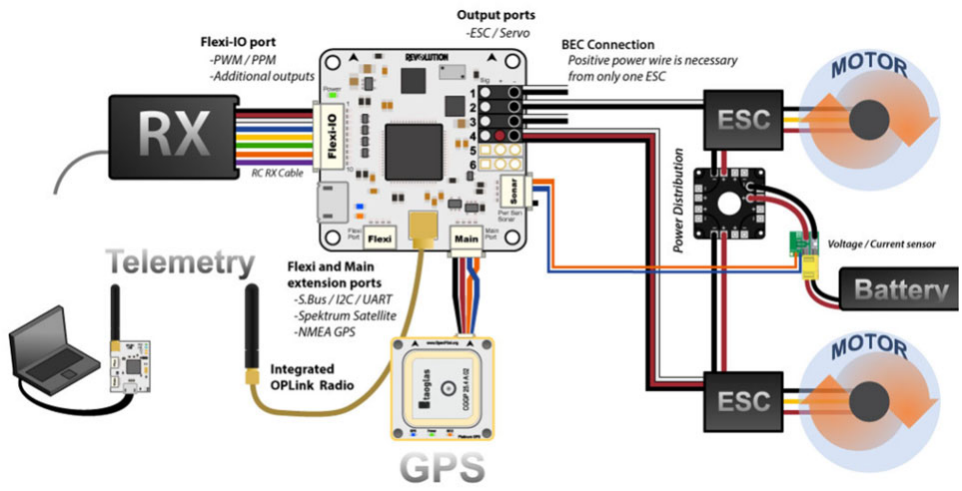
UAV hardware components Picture reprinted from https://www.google.com/search?q=Cc3d++flight+controller

A UAV is controlled by an embedded computer called the Flight Control System (FCS) or flight controller [34–36], basically consisting of a control software loaded into a microcontroller. The microcontroller reads information from on-board sensors, such as accelerometers, gyroscopes, magnetometers, pressure sensors, GPS, etc.,as well as input from the pilot, perform control calculations, and control the motors on the UAV [37, 38]. The FCS as well as the set of sensors would be mounted on the drone air frame. Drone air frames, typically made of strong, light composite materials, are mostly relatively small with limited space for avionics [39, 40]. A set of sensors, such as TV cameras, infrared cameras, thermal sensors, chemical, biological sensors, meteorological sensors etc., used to gather information during drone applications need to be lightweight to reduce UAV payload [41–44]. The information gathered from the sensors can be partially processed on-board or transmitted to the ground station for further processing [45–47]. An on-board controller, separate from the flight controller, can be used to operate the payload sensors [48–50]. Figure 5 shows the Cc3d open source flight controller used as a UAV flight controller.

The Pixhawk flight controller is an open-source hardware project equipped with sensors necessary for flight control [51–53]. It includes a CPU with RAM as well as gyroscope, compass, 3-axis accelerometer, barometric pressure, and magnetometer [54, 55]. The Paparazzi flight controller, developed by Ecole Nationale de lAviation Civil (ENAC) UAV Lab since 2003 [34], is the first and oldest open-source drone hardware and software project. In March 2017 ENAC Lab released the Paparazzi Chimera autopilot. A detailed survey on open-source flight controllers was disclosed by Ebeid et.al in [34]. An autopilot software is used for drone automatic flight control [56]. On the other hand, drones can be operated remotely through a remote controller [57–59].

### State observation

The FCS requires information on UAV states such as attitude, position, and velocity for control implementation [60]. The commonly used state observer is the inertial guidance system. Other attitude determination devices such as infrared or vision based sensors can be used [61, 62]. The inertial guidance system (IGS), also referred to as inertial navigation system (INS) [63] consists of the inertial measurement unit (IMU) and the navigation computer. The IMU has three orthogonal rate-gyroscopes, three orthogonal accelerometers and sometimes 3-axis magnetometer to determine angular velocity, linear acceleration and orientation respectively [64]. Inertial guidance systems are entirely self reliant within a vehicle where they are used. They do not rely on transmission of signals from the vehicle or reception of signals from external sources. Inertial guidance systems can be used to estimate the location of the UAV relative to its initial position using a method known as dead reckoning [65]. Global navigation satellite system (GNSS) provides location estimates using at least four satellites [65].

### State estimation

State estimation feedback is required for UAV control, such estimates are usually for attitude, position, and velocity [66]. On board sensor readings are fed to the UAV autopilot system to generate UAV state estimates [67]. The need for state estimation is due to the fact that data from measurement sensors is prone to uncertainties due to atmospheric disturbances, vibrations noise, inaccuracy of coordinate transformations, and missing measurements [68]. Sensors such as the GPS suffers from signal obstruction and reflections caused by nearby objects leading to missing or inadequate information [69].

To compensate for uncertainties and lack of information from individual sensors, multiple sensor data fusion can be employed to incorporate advantages of different types of sensors [70]. The altitude heading and reference system combines gyroscope, accelerometer, magnetometer, GPS and pressure sensors to measure UAV states. Sensor data for state estimates need to be updated at a relatively high frequency, normally above 20 Hz for small UAVs. Kalman filtering can be employed to make optimal estimations for sensors with lower update frequencies, such as the GPS, which typically has an update frequency of 4 Hz. Kalman filtering can also be used to process gyroscope readings which are susceptible to noise and drift. The other technique to improve gyroscopic readings is to model the gyroscope random noise and then offsetting it according to the model, this is referred to as model compensation [71].

### Controller design for autopilots

Most current commercial and research autopilots focus on GPS-based waypoints navigation to follow a desires path [72]. Waypoint navigation is essential for autonomous control of UAVs for UAV tasks beyond the pilot’s sight. The pilot could control the UAV from the GCS using a graphical User Interface (GUI), the location as well as other needed information about the UAV would be displayed at the the GCS [45]. The path following control of a UAV involves the control of roll, pitch, altitude and air speed for trajectory tracking and waypoint navigation [73]. GPS waypoint navigation involves providing sequential GPS coordinates that contains locations and heights of the UAV flight [72]. The set of pr-programmed GPS waypoints then becomes the path for the UAV to follow [74]. In

### Microcontrollers used

An FCS has sensor packages for state determination, on-board processors for control and estimation uses, and peripherals for communication links and data transfer. For small UAV applications , small, light weight, and often low power consumption hardware components for the FCS are preferable. Successful UAV control requires sensors used for attitude estimation to have good performance especially in mobile and temperature-varying environments [75]. Arduino is an open-source electronics platform found in a wide variety of application projects. The board is capable of reading inputs from various sensors and generates required outputs. It comes comes with different processors and board sizes. Arduino Nano was used in [76] to develop an instrumentation system to collect flight data such as airspeed, orientation, and altitude, e.t.c. The system will then transmit the flight data over a radio frequency module.

### Rotors configuration

**Fig. 6:**
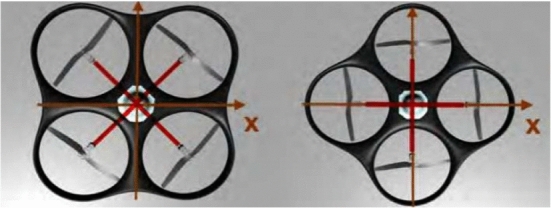
Quadrotor cross and plus Configuaration Picture reprinted from [82]

There are different types of drones, they can generally be categorised as single rotor helicopter, fixed wing and multi-rotor drones [77, 78]. Nowadays researchers endeavors to combine the advantages of fixed wing and multi-rotor drones [77]. Fixed wing drones are renowned for their endurance whereas helicopters and multirotors have the the advantage of VTOL as well as hovering. Quad-rotor drones are most common and belongs to the multi-copter family [77]. The quad-rotor unmanned aerial vehicle (UAV) are drones with four rotors typically designed in a cross configuration with two pairs of opposite rotors rotating clockwise and the other rotor pair rotating counter-clockwise to balance the torque. The roll, pitch, yaw and up-thrust actions are controlled by changing the thrusts of the rotors using pulse width modulation (PWM) to give the desired output [79]. Typically, the structure of a quad-rotor is simple enough, which comprises four rotors attached at the ends of arms under a symmetric frame. The dominating forces and moments acting on the quadrotor are given by rotors, driven with motors, mostly brushless DC motors. There are two basic types of quad-rotor configurations; plus and cross configurations [80]. The difference between these configurations is where the front of the quadcopter is located. To counteract reactional torque due to propeller rotation, two diagonal pair of motors (1 and 2) rotate anticlockwise while the other pair, motors (3 and 4), rotate clockwise [80]. In contrast to the plus configuration, for the same desired motion, the cross-style provides higher momentum which can increase the maneuverability performances, each move requires all four blades to vary their rotation speed [81]. However, the attitude control is basically analogous. Figure 6 shows the quadrotor cross and plus configurations respectively. The red cross depicts direction to the front of the quadrotor, in this case to the right of the pictures in the figure.

The quad-rotors translational motion depends on the tilting of rotor craft platform towards the desired orientation. Hence, it should be noted that the translational and rotational motion are tightly coupled because the change of rotating speed of one rotor causes motion in three degrees of freedom. This is the reason that allows the quad-rotor with six degrees of freedom (DOF) to be controlled by four rotors; therefore the quad-rotor is an under actuated system [83]. In principle, a quad-rotor is dynamically unstable and therefore proper control is necessary to make it stable. Despite the unstable dynamics, it has good agility. The instability comes from the changing rotor craft parameters and the environmental disturbances such as wind. In addition, the lack of damping and the cross-coupling between degrees of freedom make it very sensitive to disturbances.

### Sensors used

Essential to drone flight is the Inertial Guidance System, this is an electronic system that continuously monitors position, velocity and acceleration by means of incorporated sensor set. It consists of 3-axis rate gyro and 3-axis accelerometer as well as a magnetometer. The IGS readings are filtered to estimate the attitude of the UAV. Recent developments in computing and MEMs technology has seen the decrease in size of IGS sensors [84]. Thus for small UAVs, a micro IGS can be used to provide a complete set of sensor readings [75]. Attitude information can also be estimated using infrared (IR) thermopile sensors. They work on the fact that the earth emits more IR than the sky by measuring the heat difference between two sensors on one axis to determine the angle of the UAV. Other sensors such as Vision sensors, either by themselves or combined with inertial measurements sensors can also be used for attitude estimation [85].

## Required software components for real-time implementation

Real-time control requires hardware and software systems to be implemented together. Several definitions for real-time systems can be found in the literature. A good definition that we found states that; “a real-time system is one in which the correctness of a result not only depends on the logical correctness of a calculation but also upon the time at which the result is made available” https://www.ibm.com. There is a time requirement, referred to as a deadline, under which the system tasks must be performed. The primary objective is to ensure a timely and deterministic response to events. In the context of drone control, such tasks are normally intended to react to external events in real-time. Thus such real-time tasks are required to keep up with external changes affecting drone performance. Tasks required to meet their deadlines to avoid catastrophic consequences are called hard real-time tasks. When meeting the deadline is desirable but not mandatory, the task is considered soft real-time task [86].

### Real-time operating systems

A real-time operating system (RTOS) provides services such as multitasking, scheduling, inter-task communication, etc., to facilitate the implementation of real time-time systems [87]. An RTOS is the key component needed to build a real-time system. Other software pieces such as compilers, linker, debugger and drivers are necessary to interface with system hardware: https://www.ni.com. RTOSs are employed in the development of many applications such as Internet of Things (IoT), automotive , medical suystems, robotics, industrial automation, avionics, and flight control systems [88, 89]. RTOSs mainly focus on task predictability and efficiency, therefore have features to support timing constraints for application tasks [90]. There are several categories of RTOS; small, proprietary kernels as well as real-time extensions to commercial time-sharing operating systems such as Unix and Linux. The kernel is the core, an essential center of the RTOS, or any computer operating system. It is responsible for memory management, processing, and task management, and to interface with hardware and application software. Small, proprietary kernels are often used in embedded applications when very fast and highly predictable execution must be guaranteed. Meeting time constraints requires kernels to be small in size, which reduces RTOS overhead. Kernels must also have a fast context switch, support for multi-tasking, priority-based preemption, provide a bounded execution time for most primitives, and maintain a high-resolution real-time clock [90].

### Scheduling and prioritisation

Appropriate task scheduling in real-time applications is the basic mechanism adopted by an RTOS to meet time constraints of tasks [90]. It is the responsibility of the application developer to choose an RTOS that will schedule and execute these tasks to meet their constraints. For a given application, if a set of tasks can be scheduled such that they all meet their deadline, then the tasks are said to schedulable [91] In priority-driven (PD) scheduling, priorities are assigned to tasks. A task with the closest deadline than any other task is considered the highest priority task [92]. Embedded time critical applications employ the real-time scheduler to ensure low latency and meeting time constraints. Numeric priorities are assigned to threads constituting tasks, and only the highest priority task is selected to run by the scheduler. A higher priority task can preempt a lower priority task at any point of its execution [93].

However task priorities can also be dynamic such that a low priority task may temporary elevates its priority to prevent interruption during execution of its critical section. Preemption thresholds can also be set by considering task priority as well as task urgency. Both priority and Urgency are quantified such that it is possible for urgency to take precedence when scheduling tasks [86, 93]. Multithreaded parallel programming systems (MPPS) has a characteristic that data is shared among threads. It is important that access to shared data is controlled to avoid associated concurrency errors. As an example, suppose a task alters or updates a global variable, it is necessary for the task to have exclusive access to that variable while it is executing, otherwise concurrent access to the same variable by other tasks will lead to data races, leading to miscompilations.Access of shared data by one task at a time can be achieved by use of Mutual exclusion locks (mutexes) [93].

### Sensor inputs and feedback control

**Fig. 7:**
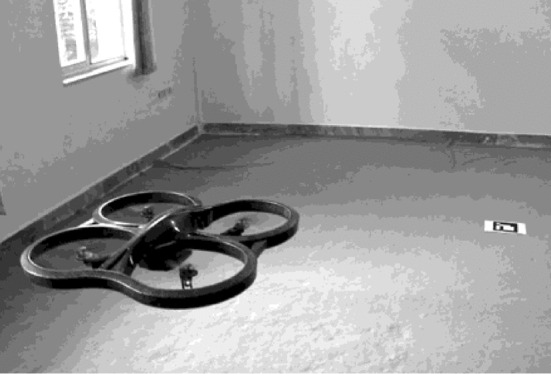
Automatic navigation and landing of an AR drone quadrotor Picture reprinted from [96]

The common drone platform has a specialised software running on a computer at the ground control station. It allows users to monitor and send control messages to affect drone’s state and actions remotely. Aboard the drone, the autopilot software combines operator inputs and sensor feedback information to directly control UAV actuators [94]. Sensors onboard the UAV provide feedback data essential to determine the drone’s position and attitude. A stereo camera was proposed for obstacle avoidance as well as velocity estimation in [95]. In [96], vision and IMU sensors were employed for automatic navigation and landing of an AR drone quadrotor. A landing marker was positioned in the drone frontal camera’s sight of view, see Fig. 7. The landing marker position is the desired position $X_d$ = ($x_{d_G}$, $y_{d_G}$, $z_{d_G}$), which corresponds to a height above the landing marker. Position $X = (x_G, y_G, z_G)$ denotes the drone current location. The position error is then denoted as $E = X_d - X$, where $E = (e_x,e_y,e_z)$. The symbols $e_x,e_y,e_z$ are position errors in directions $X_G$, $Y_G$, and $Z_G$, respectively. The PID controller was applied to the position error in accordance with (1) and (2). The drone will land when above the marker, i.e., when the error $E =0$.

1 $$\begin{aligned} V_x= & {} K_{px}e_x - K_{dx}\frac{dx_G}{dt}+ K_{ix}\int _{0}^{t}e_xdt \end{aligned}$$

2 $$\begin{aligned} V_y= & {} K_{py}e_y - K_{dy}\frac{dy_G}{dt}+ K_{iy}\int _{0}^{t}e_ydt,\quad V_z = K_{pz}e_z \end{aligned}$$

#### Localisation using differential global positioning system (DGPS)

Differential global positioning system (DGPS) is extensively used for accurate localisation of drones. The scope of localization and mapping for an agent is the method to locate itself locally, estimate its state, and build a 3D model of its surroundings, by employing among others vision sensors [97]. Towards this direction, a visual pose-estimation system from multiple cameras on-board a UAV, known as multi-camera parallel tracking and mapping (PTAM), has been presented in [98]. This solution was based on the monocular PTAM and was able to integrate concepts from the field of multi-camera ego-motion estimation. Additionally, in this work, a novel extrinsic parameter calibration method for the non-overlapping field of view cameras has been proposed.

#### Mobile phone technology in UAV applications

UAV applications encompass many areas, including, aerial surveillance ,reconnaissance, underground mine rescue operations, and so on [25, 99]. Some of these application areas are GPS denied, thus GPS can not provide the location for a UAV. Currently, vision sensors, laser scanners, and the IMU are the most common position sensors used for UAV self-localisation. In some applications, small UAVs are preferred for their cost and high maneuverability. Considering the limited load capacity and the cost of small UAVs, it cannot be equipped with sensors of high precision and large volume [100].

**Fig. 8:**
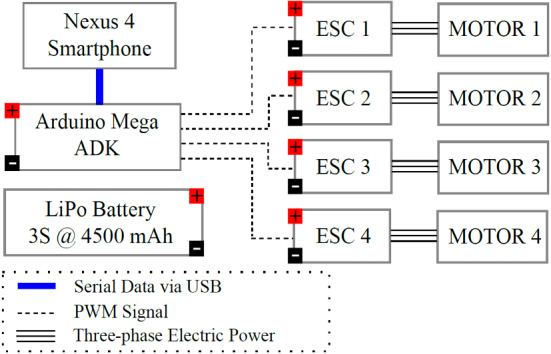
Schematic diagram for on-board smartphone flight controller using Arduino Mega to interface with the electronic speed controllers (ESCs) Picture reprinted from [101]

**Fig. 9:**
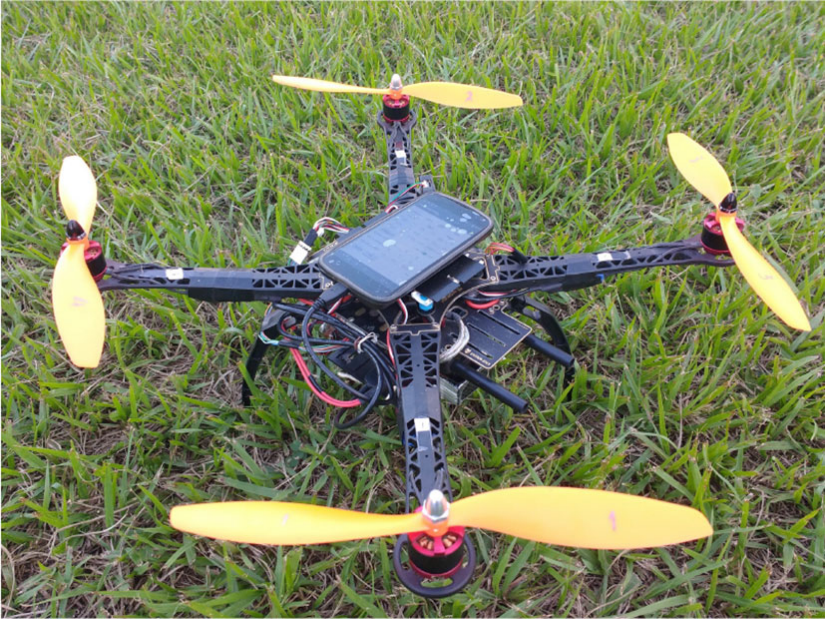
Quadcopter used in [101] with an on-board smartphone as flight controller Picture reprinted from [101]

Micro-electro-mechanical system (MEMS) sensors are therefore preferred alternatives because they are small and cheap. On the other hand, mobile phones contain multi-sensors, multi-core processors, have a small volume, and lightweight. In [101], Nexus 4 smartphone developed by Google, was used as a flight controller. The phone is equipped with inbuilt MEMS sensors such as accelerometer, gyroscope, magnetometer, global navigation satellite system (GNSS), and barometer. The implementation exclusively used sensors and processors from the smartphone, see Figs. 8 and 9. Mobile phone usage possibilities in UAV platforms are further elaborated in [102], where a smart phone is proposed for implementation of drone control algorithms. The usage of smart phones can reduce development time as it it cuts down the need for integration of different drone hardware components, instead the proposed solution uses smart phone inbuilt sensors [102].

#### Communication to the ground control station

**Fig. 10:**
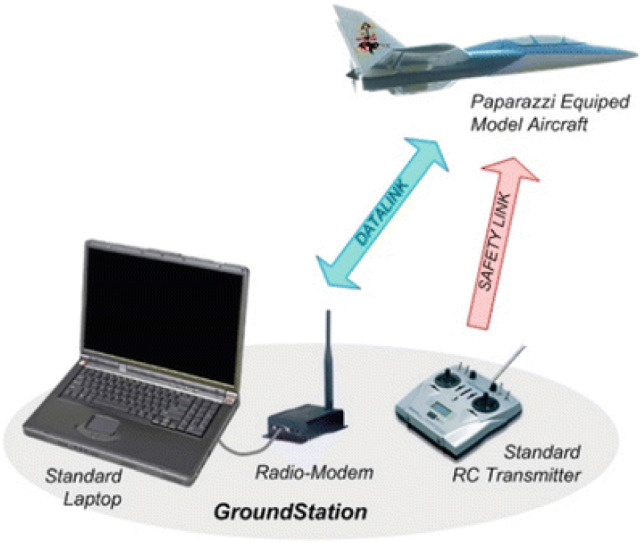
Platform for drone control from GCS Picture reprinted from https://www.google.com/search?q=multirotor+UAV++ground+control+station+images

Communication to the ground control station allows drone pilots to remotely configure mission parameters, such as coordinates to cover during way-point navigation and the action to take at each way-point. Most existing drone platforms have the configuration shown in Fig. 10. A specialized software runs at a ground-control station (GCS) to let users configure mission parameters. The Ground Control Station is a system made up of software and hardware necessary for UAV remote control. Hardware, such as the joystic, takes the pilot’s command which is transmitted to the drone via radio transmitter. The GCS software collects tellemetry data transmitted from the UAV and displays it the on the GCS user interface [103]. Communication networking is responsible for the information flow between GCS and UAV on a mission. It needs to be robust against uncertainties in the environment and quickly adapt to changes in the network topology. Communication is not only needed for disseminating observations, tasks, and control information but also needed to coordinate the vehicles more effectively toward a global goal. The goal could be tasks such as areal monitoring or detecting events within the shortest time, which are especially important in disaster situations. Some specific issues that need to be addressed [41] are connectivity, routing-and-scheduling, communication link models, and data transmission.

### Real-time scheduling algorithms

Real-time scheduling aims to complete tasks within specific time constraints and avoiding simultaneous access to resources shared amongst application tasks. To guarantee real-time performance while meeting all timing, precedence and resource usage specifications requires employment of efficient scheduling algorithms supported by accurate schedulability analysis techniques [104]. Real-time scheduling algorithms can be implemented for uniprocessor or multiprocessor systems [105–107].

In the context of drone applications, an example could be implementing a flight control system using Arduino Uno or other single processor boards. The Arduino Uno uses the ATMEGA 328P processor (uni-processor), whereas embedded computers like the Rasberry-Pi uses a quad core ARM Cortex-A72 processor (multi-processor). Scheduling algorithms can be broadly divided into two major subsets: offline scheduling and online scheduling algorithms [104]. In offline scheduling algorithms, task scheduling is carried out before system execution, also known as pre-run time scheduling. The scheduling information is then employed during run-time. The YDS algorithm (named after the author) [108], which schedules tasks according to earliest deadline first (EDF) precedence [109] is an example of an offline scheduling algorithm. By contrast, online scheduling algorithms schedule tasks at run-time.An online scheduling algorithm that encoporates event-driven and periodic rolling strategies (EDPRS) is discussed in [110].

## Types of controllers

UAV control requires an accurate and robust controller for altitude as well as velocity-and-heading [111].The altitude controller drives the UAV to fly at the desired altitude, including landing and take-off stages. The heading and velocity control enables UAV to fly through desired waypoints [112]. To achieve the above control requirements, different control strategies such as Fuzzy Logic,Linear Quadratic Regulator (LQG), Sliding Mode Control (SMC), Proportional Integral Derivative (PID), Neural Network (NN), e.t.c can be used. Robust control systems have been widely developed to address parametric uncertainties and external disturbance. In case of multirotor UAVs uncertainties arising from propeller rotation, blades flapping, change in propeller rotational speed and center of mass position dictates the need for a robust nonlinear controller [113]. In [113] robustness as well as compensation forsysten nonlinearities was adresses by combinig the nonlinear sliding mode control (SMC), robust backstepping controller and a nonlinear disturbance observer (NDO). The backstepping controller stabilised translational movement while the SMC controlled the rotational movement of the quadrotor.

The NDO provided all the estimates of disturbances ensuring robustness of the feedback controls. The PID controller was compared with a neural network controller, specifically the direct inverse control neural network (DIC-ANN) in [114]. The comparison was done in simulation, where both controllers were excited with the same reference altitude reference input and their performances plotted together.The simulation aimed to mimic a quadrotor flight in four phases comprising take-off and climb phase at $0~<~t<~10~s$, hovering phase at $10~<~t~<20~s$, climb in ramp phase at $20~<~t<~22.5~s$, and lastly the final altitude phase at $22.5~<~t<~50~s$. The comparison results showed that the DIC-ANN performed better than the PID controller in handling quadrotor altitude dynamics.Also at hovering conditions the DIC-ANN exhibited less steady state error as compared to the PID controller and the transient oscillations damped faster with the DIC-ANN showing that it handles nonlinearities better than the PID controller.

PID controllers are widely used in autopilots due to their ease of implementation, how ever they have limitations when operating in unpredictable and harsh environments. In [115] the performance of and acuracy of an attitude controller was investigated. The attitude controller is a neural network (NN) based controller trained through reinforcement learning (RL) state of the art algorithms, the Deep Deterministic Policy Gradient (DDPG), Trust Region Pocy Optimisation (TRPO), and the Proximal Policy Optimisation (PPO). The NN controller performance was compared to the performance of a PID controller to determine the appropriacy of NN controller in high precision, time-critical flight control. The contoller performance was evaluated in simulation using GYMFC environment. The results showed that RL can trail accurate attitude attitude controllers, also the controller trained with PPO outperformed a fully tuned PID controller on almost every metric.

The linear quadratic regulation (LQR) optimal control algorithm operates a dynamic system by minimizing a suitable cost function [79]. When the LQR is used with linear quadratic estimator (LQE) and Kalman filter, it is then referred to as the linear quadratic Gaussian (LQG) The LQG was applied in [116] for altitude control of a quadrotor micro aerial vehicles (MAVs). Ignoring air resistance, the linearized model for altitude control problem was obtained as (3), the state space model is represented by (4) , while the cost function is given by (5), also refered to in [116] as the quadratic form creterion. The control objective is to determine the control input *U*(*t*) to minimise cost function [79].

3 $$\begin{aligned} \ddot{Z} =a=\frac{F - mg}{m} \end{aligned}$$

4 $$\begin{aligned} \begin{aligned} \dot{x}_a(t)&=\frac{d}{dt} \left[ \begin{matrix} x(t) \\ x_r(t) \end{matrix} \right] \\&= \left[ \begin{matrix} A &{} 0 \\ -C &{} 0 \end{matrix} \right] \left[ \begin{matrix} x(t) \\ x_r(t) \end{matrix} \right] + \left[ \begin{matrix} B \\ 0 \end{matrix} \right] u(t) + \left[ \begin{matrix} 0 \\ I \end{matrix} \right] r(t) \\&= A^*x_a(t) + B^*u(t) + \left[ \begin{matrix} 0 \\ I \end{matrix} \right] r(t), \end{aligned} \end{aligned}$$

where

5 $$\begin{aligned} A^*= & {} \left[ \begin{matrix} A &{} 0 \\ -C &{} 0 \end{matrix} \right] ,~~ B^* = \left[ \begin{matrix} B\\ 0 \end{matrix} \right] .\nonumber \\ J= & {} \frac{1}{2}\int _{0}^{\infty }[x_a^TQx_a(t)+u^T(t)Ru(t)]~dt. \end{aligned}$$

**Fig. 11:**
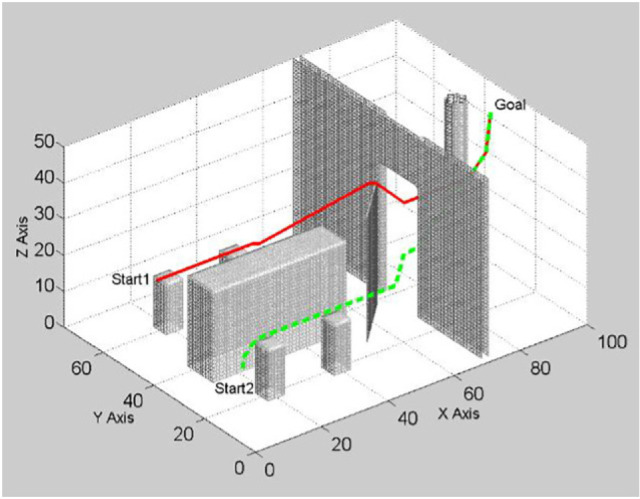
Drone path planning from start 1 and Start 2 to Goal, shortest path taken from both starting points Picture reprinted [127]

The linear Quadratic regulator with and integral with an integral term (LQTI) and a model predictive controller were employed to develop an automatic carrier landing system for a UAV [117]. The LQTI was applied to the coupled multi-input multi-output (MIMO) UAV dynamic model to reduce steady stare error while the model predictive controller was applied to the final phase landing of the UAV. Automatic carrier landing was performed sequentially by the two controllers. The LQTI controller was applied up to a few seconds before touch down followed by the MPC controller during the final stage of landing. The controller was verified via simulations on HSS Hydro toolbox. Simulation results indicated that the proposed carrier landing system can improve landing accuracy. The performance of the controllers indicted that the LQTI is suitable for calm sea environments while the MPC performs better even in rough sea environments [117]. Some implementations for UAV control employ the sliding-mode control (SMC) strategy. Sliding-mode control is a nonlinear control method that that utilises a high-frequency switching control signal to the system to command it to slide along a prescribed sliding manifold [118, 119]. It encompasses a broad range of varying fields, from pure mathematical problems to application aspects [120] (Fig. 11).

**Fig. 12:**
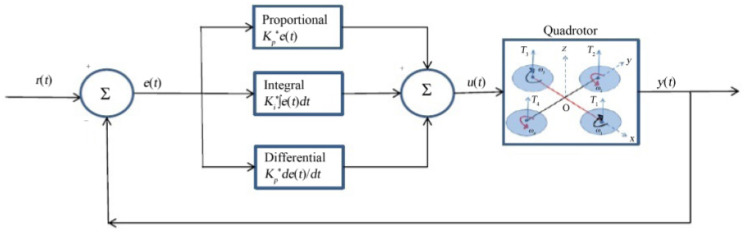
Block diagram of PID controller applied to a quadrotor [79]

**Fig. 13:**
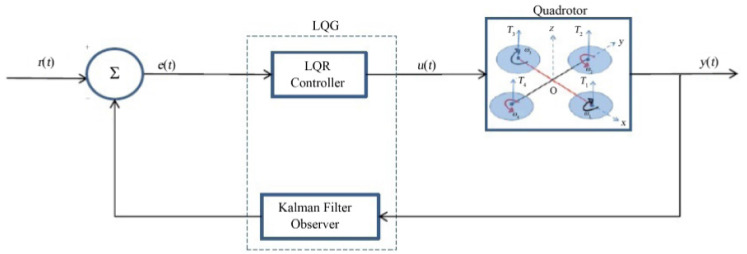
Block diagram of LQG controller applied to a quadrotor [79]

**Fig. 14:**
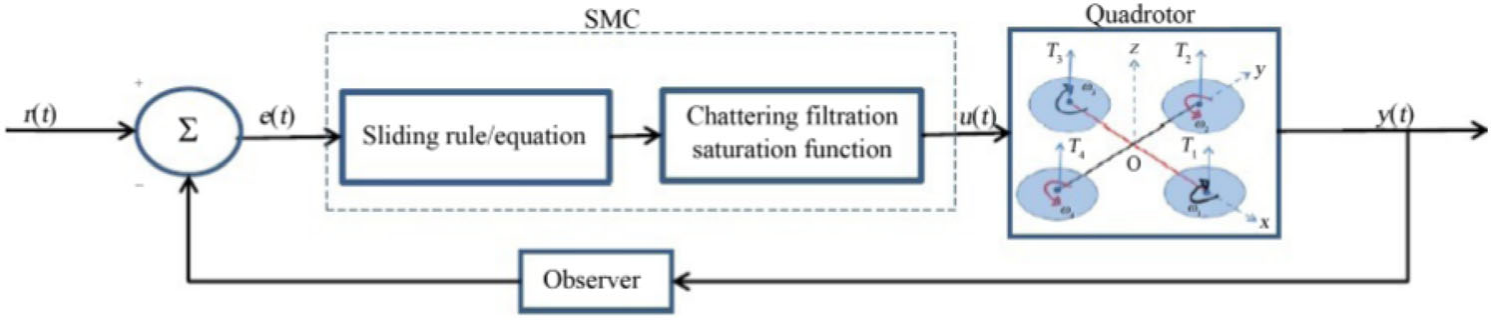
Block diagram of an SMC controller applied to a quadrotor [79]

An SMC based fault tolerant control design for underactuated UAVs was implemented on a quadrotor in [121]. The design approach separated system dynamics into two sub-systems, a fully actuated and an under-actuated subsystem. A Nonsingular Fast Terminal Sliding Mode Controller (NFTSMC) was then designed for the fully actuated subsystem, the Under-actuated Sliding Mode Controller (USSMC) was then derived for the under-actuated subsystem. The controller performance, on a quadrotor platform, demonstrated excellent robustness to actuator faults, disturbances. It had fast convergence and high precision tracking. Herrera et al. designed a sliding-mode controller and applied it in simulation of a quadrotor. They considered a PD sliding surface for vertical take-off and landing. Broad coverage of control algorithms for quadrotors can be found in [79, 122–124]. Figures 12, 13 and 14 shows the PID, LQG, and SMC controllers applied to a quadrotor respectively.

## Path planning

Missions of UAVs usually involve travelling from some initial point to a goal point [125, 126]. A mission requires generating a path for the UAV to follow. Path planning is one of the main aspects of autonomous navigation [127]. The path planning problem is to produce a path or set of waypoints for the drone to follow while taking into account the environmental and physical constraints of the drone in order to achieve a collision free flight [128, 129]. This is obstacle avoidance while executing the the UAV’s mission. Figure 11 depicts drone paths from start to goal position for two drones launched from different locations, each calculating its best path to reach the goal position.

In the literature pertinent to UAV path planning, several algorithms for measuring distances to obstacles and calculations of the drone’s path are suggested [130–132]. An optimal flight path planning mechanism to determine the best path of the UAV was developed in [133]. Consideration of environmental information such as geographical topology,location dependent wireless communication channel statistics and flight risk, sensor node deployment and worth of sensing information for different sensor types was made. The implementation aimed at determining the best path to maximise the value of gathered sensing information as well as to minimise flying time, energy consumption, and UAV operational risks. In [127], 3D propagation approximate Euclidean distance transformation algorithm was formulated to achieve safe path planning by calculating a 3D buffer around the obstacles. The algorithm prevents the drone from flying too close to obstacles by setting the minimal distance from obstacles according to the size of the drone. The algorithm is also used for drone path planning in [127]. It is worth noting that current techniques for UAVs path planning are application dependent. Different applications require different path-planning approaches.

A method to enhance massive unmanned aerial vehicles for mission critical applications (e.g., dispatching many UAVs from a source to a destination for firefighting) is investigated in [134]. The method aims to achieve UAV fast travel while avoiding inter-UAV collision while executing their mission. The path planning problem is tackled by exploiting a mean-field game (MFG) theoretic control method. The method requires UAV state exchange only once at launch, thereafter each UAV controls its acceleration by locally solving two partial differential equations, the Hamilton-Jacobi-Bellman (HJB) and Fokker-Planck-Kolmogorov (FPK) equations. Due to high computational burden posed by solving the partial differential equations, two machine learning models were used to approximate the solutions of the HJB and the FPK. The performance of the proposed method was validated on simulation, showing that the mean-field game method guarantees UAV collision avoidance. Also for the proposed approach, the effectiveness of the mean field game method is determined by the level of the HJB and FPK training.

## UAV real-time control implementation

In order to implement real-time control for UAVs, tasks have to be defined. An RTOS is required for tasks scheduling, inter-task communication, and management of available resources such memory, and power consumption [135–137]. Each task is allocated a memory space, called a stack, in the microprocessor. This is enabled by the RTOS kernel’s support for multi-threading [138, 139]. Scheduling and prioritisation of tasks, as well as the update frequency of the sensors providing essential data for task execution, ensure that application time constraints are met [140]. In [141], an embedded RTOS (RT-Thread) is applied to a quadcopter to address problems of real-time response, heavy workload and difficulty in control. Practical tests in this work indicated that quadcopter control system based on RT-Thread responded real-timely, and ensured smooth flight with a PID control algorithm.

The application tasks defined in this work are attitude information acquisition, attitude information fusion, and PID control. The latter is for quadcopter control. The application task is developed on top of RT-Thread RTOS running on STM32F407VGT6 microprocessor. The processor is equipped with high-performance ARM Cortex-M4 core with maximum system frequency of 168MHz, an FPU (floating-point unit), 1 Mbyte of flash, and 192 Kbytes of SRAM. It has peripherals such as ADC, SPI, USART, controller area network (CAN) bus, DMA, etc. High operating frequencies and high-speed memory provide high computational power to enable quadcopter complex calculations to be performed. Also additional peripherals reduce the need for external IC and reduce computational burden from the microprocessor. The implementation in [142] uses a dual processor configuration.

**Fig. 15:**
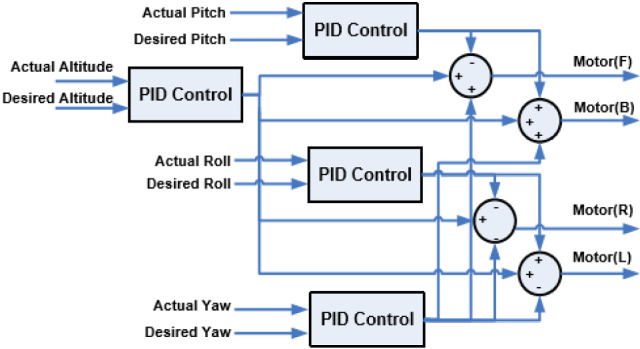
PID control loops implemented by the control processor Picture reprinted from [142]

One processor is used for telemetry and another for control of a custom quadcopter used as a test-bed. The telemetry processor executes software tasks such as communicating reconfiguration and monitoring data with the GCS, data collection from sensors, and wirelessly transmitting data to the GCS. The tasks are managed by $\mu$C/OS-II™, an RTOS. The control processor runs the PID controller algorithm for the quadcopter stabilization and navigation. This task was achieved through several tasks allocated to the control processor. Tasks include reading GPS, compass, IMU, and altitude sensor data received from the telemetry processor. Other tasks include implementation of the roll, pitch, yaw, and altitude PID control loops, and communicating reconfiguration and monitoring data with the telemetry processor via CAN bus. Figure 15 shows the PID controllers used in the implementation.

## Essential components for UAV real-time applications

### Real-time operating system (RTOS)

The literature pertaining to real-time implementation of drone control systems is relatively limited, and the number of reported studies on UAV scheduling has been minimal [143]. The main feature of real-time implementation in drones control is that an embedded RTOS, also referred to as UAV operation system in some literature, is required [67, 144]. The RTOS provides a real-time kernel on which the control program running on a micro-controller is implemented. The real-time kernel guarantees application tasks meet their time constraints by employing the UAV scheduling system [143]. Consequently, a Real-Time Operating System (RTOS) that provides operating environments for various mission services on UAVs is crucial [145]. The commonly used RTOS for UAVs is FreeRTOS, and an empirical study of this RTOS was conducted in [145]. The study looked at aspects such as functionality changes during the evolution of FreeRTOS. A total of 85 releases of FreeRTOS, from V2.4.2 to V10.0.0 were considered.

**Table 1:** Summary of Components for Real-time Control Implementation

RTOS	Hardware required	Controller used	Sensors used	References
RT-Thread	STM32F407VGT6 processor	PID	MPU-6050 (an IMU)	[141]
ERIKA Enterprise	dsPIC 30F6014 micro-controller	PID	3 gyroscopes (one for each axis); 3-axis accelerometer; inclinometer; GPS module	[148]
FreeRTOS	AVR XMEGA	PID	IMU	[149]
$\mu \hbox {C/OS-II}^{\mathrm{TM}}$	Two Freescale HCS12 microcontrollers	PID	IMU; GPS; Compass	[142]
QNX Neutrino	PC/104 (CRR3-650, Lippert); DIAMOND-MM-32-AT data acquisition board	-	Crossbow NAV420 combines GPS and IMU	[149]

1 RTOS—Real-Time Operating System; ERIKA—Embedded Real tIme Kernel Architecture; $\mu {C/OS-II}$—Micro Controller Operating System-II

### Microcontroller

The microcontroller is the UAV onboard processing unit for UAV computations and UAV state monitoring [146, 147]. It is selected such that it matches application task requirements. Considerations such as computational speeds and communication with onboard sensors have to be made. Palossi et al. [146] extended the hardware and software of a 27 grams nano-size, commercial off-the-shelf (COTS) quadrotor, the crazyflie 2.0, to achieve object tracking capability. The quadrotor platform consists the STM32F405 microcontroller as the main onboard processing unit, the Nordic nRF51 module for wireless communication. The STM32 is an ARM Cortex-M4F microcontroller, operating at 168MHz. The on-board sensing is performed by a 9-axis IMU, the MPU-9250 with a gyroscope, an accelerometer, a magnetometer, and an ST LPS25H pressure sensor with a typical accuracy of $\pm 1$ meter. The vehicle is powered by a 240mAh Li-Po battery.

**Fig. 16:**
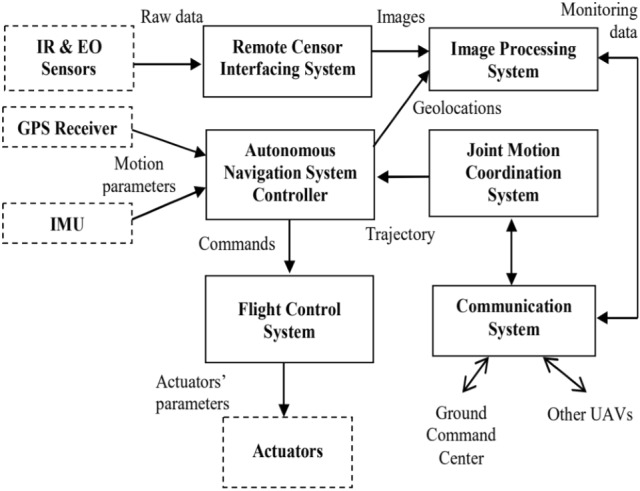
Sensors connected to microcontroller Reprinted from [150]

### Sensors and actuators

In UAV applications several sensors and actuators are connected to the microprocessor for UAV control. Table1 highlights the vital components for real-time implementation of UAV control, the table also lists various sensors used. Figure 16 shows the UAV onboard sensors used in a fire fighting remote-sensing system in [150]. Various sensors as well as the overall connection network is depicted.

## Conclusion

Real-time control of drones requires an embedded RTOS for implementation. The RTOS provides facilities such as multi-threading, scheduling and priority assignment. These support real-time response of the drone control system to feedback from GPS and IMU. The drone control system subsequently apply the corresponding motor speeds to achieve the desired drone’s movements. Multitasking enables tasks, such as position and orientation feedback, path-planning, and control implementation to run in parallel. This facilitates real-time response of the drone. Tasks may need results from other tasks for their computations. Scheduling and prioritisation of tasks ensures that at any point in time critical tasks are given computational resources by the microprocessor. For example obstacle avoidance is the highest priority task to ensure that the drone does not collide with other drones as well as other obstacles.
